# Diverting colostomy is an effective procedure for ulcerative chronic radiation proctitis patients after pelvic malignancy radiation

**DOI:** 10.1186/s12893-020-00925-2

**Published:** 2020-11-03

**Authors:** Xiaoyan Huang, Qinghua Zhong, Huaiming Wang, Jie Zhao, Yingyi Kuang, Qi Guan, Yanjiong He, Qiyuan Qin, Hui Wang, Tenghui Ma

**Affiliations:** 1 Guangdong Institute of Gastroenterology, Sun Yat-Sen University Sixth Affiliated Hospital, Guangzhou, 510655 Guangdong China; 2 Guangdong Provincial Key Laboratory of Colorectal and Pelvic Floor Diseases, Sun Yat-Sen University Sixth Affiliated Hospital, Guangzhou, 510655 Guangdong China; 3 Department of Colorectal Surgery, Sun Yat-Sen University Sixth Affiliated Hospital, No. 26 Yuancun Erheng Road, Guangzhou, 510655 Guangdong China; 4 Department of Endoscopic Surgery, Sun Yat-Sen University Sixth Affiliated Hospital, No. 26 Yuancun Erheng Road, Guangzhou, 510655 Guangdong China

**Keywords:** Chronic radiation proctitis, Rectal ulcer, Colostomy, Fistula, Influence factor

## Abstract

**Background:**

Chronic radiation proctitis (CRP) with rectal ulcer is a common complication after pelvic malignancy radiation, and gradually deteriorating ulcers will result in severe complications such as fistula. The aim of this study was to evaluate effect of colostomy on ulcerative CRP and to identify associated influence factors with effectiveness of colostomy.

**Methods:**

Between November 2011 to February 2019, 811 hospitalized patients were diagnosed with radiation-induced enteritis (RE) in Sun Yat-sen University Sixth Affiliated Hospital, among which 284 patients presented with rectal ulcer, and 61 ulcerative CRP patients were retrospectively collected and analyzed.

**Results:**

The overall effective rate of colostomy on ulcerative CRP was 49.2%, with a highest effective rate of 88.2% within 12 to 24 months after colostomy. 9 (31.1%) CRP patients with ulcers were cured after colostomy and 12 (19.67%) patients restored intestinal continuity, among which including 2 (3.3%) patients ever with rectovaginal fistula. 100% (55/55) patients with rectal bleeding and 91.4% (32/35) patients with anal pain were remarkably alleviated. Additionally, multivariable analysis showed the duration of stoma [OR 1.211, 95% CI (1.060–1.382), *P* = 0.005] and albumin (ALB) level post-colostomy [OR 1.437, 95% CI (1.102–1.875), *P* = 0.007] were two independent influence factors for the effectiveness of colostomy on the rectal ulcer of CRP patients.

**Conclusions:**

Colostomy was an effective and safe procedure for treating rectal ulcer of CRP patients, and also a potential strategy for preventing and treating fistula. Duration of stoma for 12–24 months and higher ALB level could significantly improve the effectiveness of colostomy on ulcerative CRP patients.

## Background

Radiotherapy is an essential therapeutic tool for pelvic malignancies such as uterine cervix, uterine corpus, prostate, testicular, urinary bladder and rectal cancers. Chronic radiation proctitis (CRP) is an unavoidable and commonly observed side effect, occurs 3 months later and in 5–20% of patients after pelvic malignancy radiation [1, 2]. While acute radiation proctitis (ARP) occurs within the first 3 months after radiotherapy, is transient and self-healing, CRP is usually progressive and irreversible [3, 4]. The primary clinical manifestations of CRP are diarrhea, bleeding, anal cramping pain, tenesmus, strictures, deep ulcers and fistulas [5, 6]. Endoscopic examination is the most important tool for diagnosis of CRP and the optimal method for monitoring the intestinal changes of CRP. Under endoscopy, CRP is characterized by mucosal fragility, pallor, spontaneous bleeding, telangiectasias, mucosal edema, strictures, fistulas, and ulcerations [7, 8]. Rectal ulcer is a common complication of CRP, persistent and gradually deteriorating ulcer will lead to severe complications, such as perforation, necrosis, abscess, fistulas, strictures, or even death, seriously impairing patients’ quality of life [5, 9]. In further, due to radiotherapy, the ulcer of CRP patients has a poor wound healing [10]. Until now, researches on treating ulcer of CRP are limited and require more attention.

Colostomy is preferred by some surgeons for radiation induced deep ulceration, fistula or stricture in intestine, which could prevent further deterioration of the complications and help to avoid further interventions [11, 12]. Meanwhile, for CRP patients, colostomy is able to not only obviously control rectal bleeding, but also significantly relieve the pain, dramatically relieving the suffering of patients [13]. In addition, Piekarski et al. demonstrated that rectovaginal fistula of 3 CRP patients in total 17 cases were spontaneous healed after colostomy [14]. And in clinic, we found that rectal ulcers of some CRP patients were improved after colostomy. However, data on effect of colostomy on ulcerative chronic radiation proctitis after pelvic malignancy radiation are lacking.

Therefore, in this study, effect of colostomy on the rectal ulcer of CRP patients was evaluated, and its influencing factors were analyzed as well.

## Methods

### Patients

Patients diagnosed with ulcerative CRP in Sixth Affiliated Hospital, Sun Yat-Sen University from November 2011 to February 2019 were retrospectively included. The inclusion criteria were: patients diagnosed with radiation-induced enteritis (RE) after pelvic malignancy radiation; patients with rectal ulcer under endoscopic examination; patients received diverting colostomy treatment because of fistula, intractable bleeding or unbearable anal pain; patients underwent endoscopic examination before and after diverting colostomy. The exclusion criteria were: patients with ARP; patients with incomplete clinical data.

A regular follow-up was scheduled every 6 months. A comprehensive assessment including endoscopy, magnetic resonance imaging (MRI) and defecography would be performed at 12 months to evaluate the possibility of stoma reversion. If the patients deteriorated to develop unbearable symptoms such as anal pain or severe fistula, they could choose to receive surgical resection of the involved bowel segment after being evaluated.

The clinical data such as demographics, radiotherapy dosimetry and treatments were carefully collected. This study was approved by the Ethical Committee of the Sixth Affiliated Hospital of Sun Yat-Sen University and was conducted in according with the provisions of the World Medical Association’s Declaration of Helsinki of 1995 (revised in Tokyo, 2004). Informed consent was waived, because this was a retrospective study.

### Measures and evaluation

Diverting colostomy was performed as described in previous research [15]. Briefly, after general anesthesia, the transverse colon was pulled out through a small incision, and a double-cavity stoma of the transverse colon was then created.

All the included CRP patients have performed endoscopy before and after colostomy. The severity of intestinal lesions was evaluated according to Vienna Rectoscopy Score (VRS) system from five aspects (mucosal congestion, telangiectasia, ulceration, stenosis, necrosis). A higher score indicated a more severe lesion. In detail for evaluation of the ulcer, grade 0 refers to no ulcer; grade 1 refers to small ulcer with or without surface < 1 cm^2^; grade 2 refers to ulcer area > 1 cm^2^; grade 3 refers to deep ulcer; and grade 4 refers to deep ulcer with fistula or perforation. Grade 1, grade 2 and ≥ grade 3 ulcers were scored 3, 4 and 5, respectively.

Effect of diverting colostomy on ulcerative CRP was determined by comparison of ulcer grade before and after colostomy under endoscopy. The treatment was defined as effective if the ulcer was healed or alleviative after colostomy, while ineffective if the ulcer was unchanged or aggravated. Then the included patients were divided into effective and ineffective groups to access the associated influence factors for the effect of colostomy on ulcerative CRP.

### Statistical analysis

SPSS software, version 19.0 (Chicago, IL, USA) was used for data analysis. Continuous variables were performed using the t tests or the Mann–Whitney U tests as appropriate; Categorical variables were compared using Fisher’s test or the χ^2^ test as appropriate. Univariate and multivariate analysis were used to access the associations between effective and ineffective groups for the ulcerative CRP patients. A two-sided *P* < 0.05 was considered statistically significant.

## Results

### Effect of diverting colostomy on ulcerative CRP

**Fig. 1:**
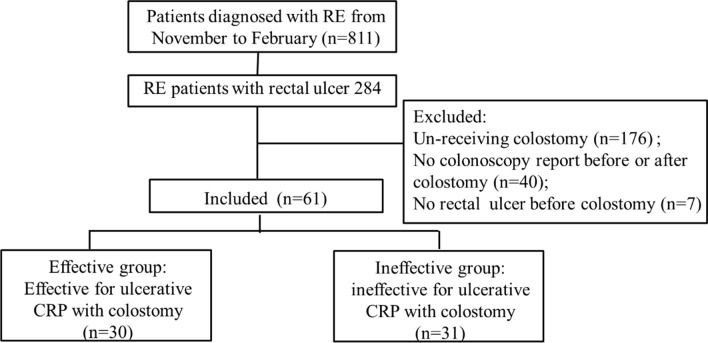
Flow chart of patient selection. RE, Radiation enteritis; CRP, Chronic radiation proctitis

From November 2011 to February 2019, there were 811 RE patients treated in our hospital, among which 284 (35%) patients presented with rectal ulcer. According to the above-mentioned eligibility, a total of 61 patients were identified to be eligible for this study, as shown in Fig. 1. The primary malignancies treated with radiotherapy were cervical cancer (n = 56), prostatic cancer (n = 2), endometrial cancer (n = 2) and rectal cancer (n = 1). The median interval between radiotherapy and colostomy was 11.8 months (interquartile ratio 8.7–16.1). The mean age at the time of colostomy was 56.5 ± 10.4 year old.

Table 1 showed the effect of diverting colostomy on rectal ulcer of CRP patients. Before colostomy, the ulcers were categorized as grade 1 in 6 patients, grade 2 in 16 patients, grade 3 in 24 patients and grade 4 in 15 patients. After colostomy, the ulcers were remarkably improved (Table 1). The effective rate was 49.2% (30/61), with 19 patients healed and 11 relieved. In the ineffective group, 11 (18.0%) patients had deep ulcers (grade 3) before colostomy, 7 of them developed fistula after colostomy. And 10 (16.4%) patients were with fistula before colostomy, 6 (9.8%) patients with ulcer of grade 2 had no significant change, and 2 (3.3%) patients with ulcer of grade 1 and 1 (1.6%) patient with grade 2 deteriorated. As for the patients ineffective with colostomy, 6 patients subsequent received surgical resection of the involved bowel segment because of unbearable pain or simultaneous with fistula, other patients did not received any further intervention as quality of life was not affected.

**Table 1:** Effect of colostomy on rectal ulcer of ulcerative CRP

Effect, n		Ulcer Grade								
		Pre-colostomy				Post-colostomy				
		1	2	3	4	0	1	2	3	4
Healed	19	4	8	5	2	19	0	0	0	0
Alleviative	11	0	1	7	3	0	5	4	1	1
Unchanged	20	0	6	4	10	0	0	6	4	10
Aggravated	11	2	1	8	0	0	0	2	1	8
Effective rate	49.2%									

Table 2 showed effect of diverting colostomy on the VRS and blood parameters of the ulcerative CRP patients. We found that the scores of mucosal congestion, telangiectasia, ulcers and stenosis obviously decreased after colostomy (*P* < 0.05). The levels of hemoglobin (Hb) (105.3 ± 18.4 g/L vs. 92.9 ± 23.2 g/L, *P* < 0.001) and red blood cell (RBC) (3.8 ± 0.6 × 10^12^/L vs. 3.4 ± 0.7 × 10^12^/L, *P* = 0.001) increased significantly, while blood platelet count (BPC) (255.5 ± 104.1 vs. 289.8 ± 105.3, *P* = 0.031) decreased compared to pre-colostomy. The result indicated that colostomy treatment not only relieved the rectal ulcer, but also improved anemia status.

**Table 2:** Comparisons between before and after colostomy on ulcerative CRP patients

	Pre-colostomy	Post-colostomy	*P*
VRS*	5 (4, 5)	5 (4, 5)	0.001^5^
Mucosal congestion*	2 (2, 2)	1 (1, 2)	0.000^5^
Telangiectasia*	2 (2, 2)	2 (1, 2)	0.002^5^
Ulceration*	5 (4, 5)	4 (0, 5)	0.000^5^
Stenosis*	0 (0, 4)	0 (0, 4)	0.032^5^
Necrosis*	0 (0, 0)	0 (0, 0)	0.317^5^
Hb, g/L^#^	92.9 ± 23.2	105.3 ± 18.4	0.000^4^
RBC, × 10^12^/L^#^	3.4 ± 0.7	3.8 ± 0.6	0.001^4^
BPC, × 10^9^/L^#^	289.8 ± 105.3	255.5 ± 104.1	0.031^4^
ALB, g/L^#^	37.5 ± 4.9	38.5 ± 4.7	0.169^4^
TP, g/L^#^	66.5 ± 7.0	68.6 ± 6.7	0.088^4^
WBC, × 10^9^/L^#^	6.9 ± 3.1	6.1 ± 2.8	0.114^4^

*VRS* Vienna Rectoscopy Score, *IQR* interquartile range, *SD* standard deviation, *Hb* hemoglobin, *RBC* red blood cells, *BPC* Platelet count, *ALB* albumin, *WBC* white blood cells

^1^Data were calculated using χ^2^ test

^2^Data were calculated using χ^2^ test with corrections for continuity

^3^Data were calculated using Fisher’s test

^4^Data were calculated using t tests

^5^Data were calculated using Mann–Whitney U tests test

^#^Values which were normally distributed were expressed as means ± SDs

*Values which weren’t normally distributed were expressed as medians (IQR)

### Comparison of the demographic and clinical characteristics between effectively and ineffectively treated patients

As Table 3 showed, the two groups of patients were comparable for baseline scores of mucosal congestion, telangiectasia, ulceration, stenosis and necrosis. Compared to the ineffective group, patients in the effective group had much longer duration of stoma, higher levels of pre-albumin (ALB), post- RBC, post- Hb and post- ALB. In contrast, there was no significant difference in age, BMI, cardiovascular and cerebrovascular disease, surgical history, radiotherapy dose, tumor recurrence or metastasis between the two groups. Interestingly, we found that levels of RBC, Hb, ALB, TP increased, and WBC decreased after the patients receiving colostomy treatment in the two groups, despite no significant difference in these factors were observed.

**Table 3:** Comparison of the demographic and clinical characteristics between effectively and ineffectively colostomy treated ulcerative CRP patients

Variables	Effective (n = 30)	Ineffective (n = 31)	*P*
Age, < 50/ ≥ 50 years	8/22	10/21	0.632^1^
BMI < 18.5/ ≥ 18.5 kg/m^2^	8/22	7/22	0.824^1^
Concomitant disease			
Cardiovascular and cerebrovascular diseases, yes/no	6/24	5/26	0.694^1^
Diabetes mellitus, yes/no	5/25	1/30	0.078^2^
Surgical history, yes/no	11/19	11/20	0.923^1^
Tumor recurrence or metastasis, yes/no	2/28	2/29	1.000^2^
Duration of stoma, M*	13.8 (9.2, 19.0)	5.8 (3.0, 8.6)	0.000
Radiotherapy dose, Gy^#^	85.2 ± 24.0	84.7 ± 20.0	0.941^4^
Interval between radiotherapy and colostomy, M*	12.3 (9.2, 17.6)	10.6 (8.5, 14.8)	0.207^5^
APC treatment after colostomy, yes/no	3/27	4/27	1.000^2^
Retention enema after colostomy, yes/no	14/16	16/15	0.699^1^
Pre- colostomy			
Overall VRS Score*	5 (4, 5)	5 (5, 5)	0.246^5^
Mucosal congestion*	2 (2, 2)	2 (1, 2)	0.204^5^
Telangiectasia*	2 (2, 3)	2 (2, 2.25)	0.678^5^
Ulceration*	5 (4, 5)	5 (4, 5)	0.259^5^
Stenosis*	0 (0, 4)	0 (0, 4)	0.804^5^
Necrosis*	0 (0, 0)	0 (0, 0)	0.283^5^
RBC, × 10^12^/L^#^	3.3 ± 0.7	3.5 ± 0.8	0.357^4^
Hb, g/L^#^	92.1 ± 23.8	91.8 ± 22.6	0.955^4^
BPC, × 10^9^/L*	265.8 (230.8, 345.0)	261.8 (208.0, 315.5)	0.882^5^
ALB, g/L*	38.0 (35.1, 42.2)	34.6 (32.2, 41.3)	0.050^5^
TP, g/L^#^	38.3 ± 4.7	35.8 ± 5.1	0.731^4^
WBC, × 10^9^/L*	5.7 (4.3, 6.7)	6.3 (4.9, 10.0)	0.106^5^
Post-colostomy			
RBC, × 10^12^/L^#^	4.0 ± 0.4	3.5 ± 0.8	0.009^4^
Hb, g/L^#^	112.0 ± 15.6	98.7 ± 19.0	0.006^4^
BPC, × 10^9^/L*	233.5 (187.5, 277.5)	241.0 (198.8, 320.7)	0.463^5^
ALB, g/L^#^	40.5 ± 3.9	36.5 ± 4.5	0.000^4^
TP, g/L^#^	68.5 ± 6.8	69.5 ± 6.7	0.597^4^
WBC, × 10^9^/L*	4.9 (4.3, 5.8)	5.6 (4.3, 8.5)	0.244^5^

*VRS* Vienna Rectoscopy Score, *IQR* interquartile range, *SD* standard deviation, *Hb* hemoglobin, *RBC* red blood cells, *BPC* Platelet count, *ALB* albumin, *WBC* white blood cells

^1^Data were calculated using χ^2^ test

^2^Data were calculated using χ^2^ test with corrections for continuity

^3^Data were calculated using Fisher’s test

^4^Data were calculated using t tests

^5^Data were calculated using Mann–Whitney U tests test

^#^Values which were normally distributed were expressed as means ± SDs

*Values which weren’t normally distributed were expressed as medians (IQR)

The univariate analysis showed statistically significant associations between stoma effectiveness and duration of stoma, pre- ALB, post- ALB, post-Hb and post-RBC. Further multivariate analysis (Table 4) showed that stoma effectiveness was independently associated with duration of stoma [OR 1.211, 95% CI 1.060–1.382, *P* = 0.005] and levels of post-ALB [OR 1.437 95% CI 1.102–1.875, *P* = 0.007].

**Table 4:** Multivariate logistic regression analysis for effectiveness of colostomy on ulcerative CRP patients

Variables	OR	95% CI	*P*
Duration of stoma	1.211	1.060–1.382	0.005
Pre-colostomy			
ALB	1.055	0.893–1.245	0.530
Post-colostomy			
ALB	1.437	1.102–1.875	0.007
Hb	1.010	0.945–1.089	0.767
RBC	0.666	0.120–3.690	0.642

*OR* odds ratio, *CI* confidence interval, *ALB* albumin, *Hb* hemoglobin, *RBC* red blood cells

### Effect of various duration of stoma for CRP patients

**Fig. 2:**
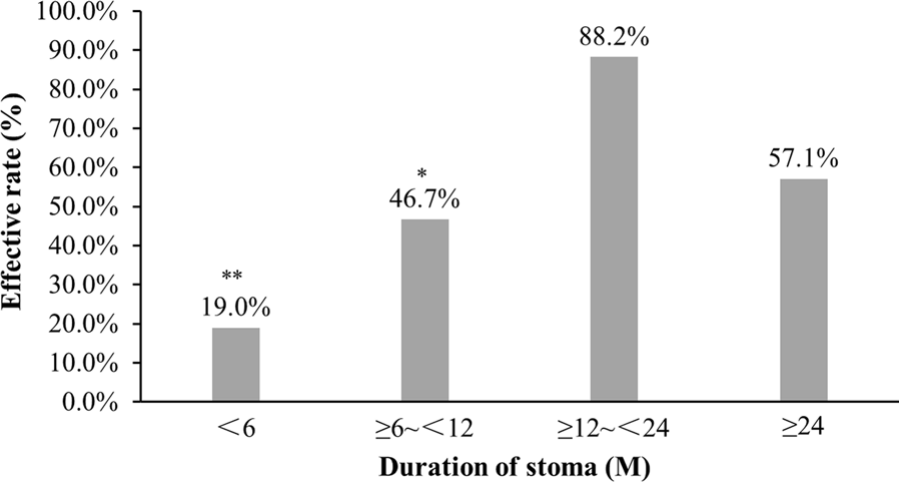
Effect of various duration of stoma for ulcerative CRP patients. The effective rate of the ulcerative CRP patients with duration of stoma of ≥ 12 to < 24 M (88.2%, 15/17) was much higher than that of < 6 M (19.0%, 4/21, *P* = 0.000) and ≥ 6 to < 12 M (46.7%, 7/15, *P* = 0.032), and has no significant difference with that of ≥ 24 M (57.1%, 4/7, *P* = 0.249). M, Months; vs. ≥ 12 to < 24 M, **P < 0.001, *P < 0.05. The effective rate of the ulcerative CRP patients with duration of stoma of ≥ 12 ~ < 24 M (88.2%, 15/17) was much higher than that of < 6 M (19.0%, 4/21, *P* = 0.000) and ≥ 6 to < 12 M (46.7%, 7/15, *P* = 0.032), and has no significant difference with that of ≥ 24 M (57.1%, 4/7, *P* = 0.249)

Figure 2 showed effect of different duration of stoma on ulcerative CRP patients. Among CRP patients with different duration of stoma, patients with stoma for ≥ 12 to < 24 months had the highest effective rate (88.2%).

### Effect on clinical symptoms and safety of colostomy

Rectal bleeding and pain were reported in 55 and 35 patients respectively. After colostomy, rectal bleeding was alleviated in all the patients (100%, 55/55); while anal pain was alleviated in 91.4% of the patients (32/35). Twelve patients had successful closure of the temporary colostomy one year after colostomy, all of them remained asymptomatic after revision. However, among the 61 included patients, 12 (19.7%) patients had stoma complications (10 prolapse and 2 parastomal hernia), and 2 patients received surgical intervention for severe prolapse.

### Evolution of fistula and treatment

We further surveyed the CRP patients with fistula performed endoscopic examination (n = 13) before fistula formation to investigate the evolution of fistula. It was observed that from grade 2 ulcers and grade 3 ulcers evolving into fistula within 4.2 (3.3, 7.1) and 3.7 (2.7, 9.8) months, respectively. Even there were 2 patients from grade 1 ulcers turning into fistula rapidly during 5.8 and 2.7 months. Among the included patients, 13 patients received colostomy because of fistula (1 in rectovaginal bladder fistula, 10 in rectovaginal fistula, 1 in rectourethral fistula and 1 in rectal fistula), 8 patients with rectal ulcer in grade 3 developing fistula after colostomy, and the remaining 40 patients without fistula before and after colostomy. In addition, it was interesting to found that the enormous suffering of the 13 patients with fistula pre-colostomy improved obviously, 30.7% (4/13) patients with fistula relieved under endoscopic observation, among which 3 patients with rectovaginal fistula were even healed and 2 patients already had their stoma closed without any discomfort 2 or 4 year later from follow-up data.

## Discussion

CRP is a quite common complication after pelvic radiation therapy, with the characteristic pathologic changes are inflammatory disease, obliterative endarteritis, fibrosis, intestinal ischemia, capillary compensatory hyperplasia, telangiectasias, resulting in ulceration, necrosis, perforation, bleeding, stricturing as the disease progresses [16, 17]. The severity of CRP is mainly depended on the volume of rectum irradiated, total radiotherapy dose, radiotherapy technique, dose per fraction, and individual sensitivity to radiotherapy [18, 19]. In this study, due to application of radiation in higher dose and brachytherapy for gynecological cancer and prostatic cancer, most of the included patients with rectal ulcer ever had cervical cancer, endometrial cancer and prostatic cancer. Ulcerative CRP patients frequently present with defecation difficulties, such as diarrhea, anal cramping pain, tenesmus, incontinence and constipation. Physiatric disorders such as worse lumbar lordosis, lower rate of perineal defense reflex and higher rate of muscle synergies presence had been demonstrated strong correlations with the symptoms of constipation and incontinence [20, 21]. However, there remains unclear that whether radiotherapy will cause these physiatric disorders. Further researches need to be conducted to investigate the relationships between these clinical and instrumental parameters and detail symptoms or ulcer sites.

Up to now, no standard guideline or procedure is established for treatment of ulcerative CRP. As for CRP, an ascending ladder therapy derived from institutional experience, case reports, and small clinical trials was adopted. Generally, the therapies broadly divided into three categories: medical therapy for mild diarrhea, mild cramping, slight pain or bleeding; endoscopic therapy for rectal bleeding, particularly those refractory to medical management; surgical therapy such as fecal diversion, repair/reconstruction and proctectomy/pelvic exenteration for more severe or refractory cases, such as refractory bleeding and pain, strictures leading to intestinal obstruction, extremely deep ulcer, fistulas, or sepsis [13, 22, 23].

Sometimes, in some patients, it was reporeted that fecal diversion improves clinic symptoms and their quality of life to the point that they do not need frequently intervention even though the underlying problem is not directly addressed [24, 25]. Moreover, diverting the fecal stream via a colostomy or an ileostomy can reduce bacterial contamination and decrease irritation injury by fecal stream, and can gain time to subside any radiation reaction to protect injured tissue [25]. And it was reported that fecal diversion cause clinical and histological remission, and normalize mucosal barrier dysfunction in patients with collagenous colitis [26–28]. Because there exist the risk of high-volume fluid discharge of ileostomy, and most of the CRP patients may require a permanent stoma, colostomy is preferable for CRP patients [29].

Up to now, whether colostomy is effective or not for ulcerative CRP is still unclear. Therefore, in this study, we evaluated the effect of colostomy on CRP patients with rectal ulcer. The included patients were all with rectal ucer, but not all received colostomy because of deep ulcers or fistula, some were mainly due to rectal bleeding. However, all the included patients presented with rectal ulcer before colostomy. Our result showed that the overall effective rate of colostomy on ulcerative CRP patients was 49.2%, which was much higher than that of the almagate compound enema (35.3%) we ever reported. Compared to pre-colostomy, the ulcer scores decreased obviously after colostomy. Interestingly, we found that duration of stoma significantly influenced effect of colostomy on the rectal ulcer of CRP, and a highest effective rate (88.2%) of colostomy on ulcerative CRP reached after the patients had stoma for 12 to 24 months. The reason may be that intestinal lesions have poor wound healing following radiotherapy leading to rectal ulcer delay to heal and apt to recur [30]. Additionally, diversion proctitis occurred between 3 and 36 months, and was more likely to appear with time after fecal diversion [31, 32]. Therefore, effective rate of colostomy on ulcerative CRP patients with stoma for ≥ 24 M (57.1%) were lower than that with stoma for ≥ 12 to < 24 M (88.2%).

In addition, after colostomy, the scores of mucosal congestion, telangiectasia and ulceration obviously decreased, all of the patients with rectal bleeding remitted, and thus level of Hb and RBC remarkably increased, which was in accordance with previous study by Yuan et al. [11]. 100% rectal bleeding remission and 91.4% anal pain alleviation achieved after the patients receiving colostomy. Our result above demonstrated that colostomy was an effective method for the rectal ulcer of CRP, and for rectal bleeding as well. However, as this is a retrospective study, severity degree of rectal bleeding, the anal pain and other clinical symptoms before and after colostomy were not prospectively evaluated. A related prospective study need to be conduct in further.

Fortunately, in this study, 12 (19.7%) CRP patients with rectal ulcer completely cured and had already closed stoma, even 3 of 13 CRP patients with fistula healed after colostomy and 2 had already restored intestinal continuity without any uncomfortable symptom 2 or 4 year later from the follow-up data. Previously, piekarski JH also reported that spontaneous closure of fistula in 3 of 17 patients occurred after fecal diversion, but no patient had her stoma closed [14]. Our result demonstrated that CRP patients with fistula had the likelihood of healing by colostomy, and also could close the abdominal stoma after the fistula being cured.

This study suggested that evolution from ulcer to fistula was rapid in some ulcerative CRP patents. Fortunately, effective rate of colostomy on ulcerative CRP was 49.2%, with fistula of 3 in13 patients cured by colostomy. But 8 patients with grade 3 of ulcer still turned into fistula, which may be because the ulcer was too deep to relieve. Colostomy could not avoid all the ulcers aggravating, depending on patient condition and ulcer depth. As the rectal ulcer is possible to deteriorate, relieve or even heal after colostomy, knowing when to choose colostomy procedure for ulcerative CRP is extraordinary important. However, the sample size of this study was small, and the ulcerative CRP patients without colostomy were not investigated, a further research was needed to clarify this issue.

While colostomy created properly can dramatically improve CRP patients’ quality of life, it also brings some complications. Colostomy-related complications including stomal ischemia/necrosis, retraction, mucocutaneous separation, parastomal abscess, parastomal hernia, prolapse, retraction, and varices were reported, ranging from 20 to 70% [33–35]. Our result showed occurrence of stoma complications was 19.7% (12/61), a little lower than that in literature. It may be because only the stoma complications of hospitalized CRP patients were investigated, its occurrence might be underestimated.

In the present study, except for duration of stoma, ALB level after colostomy was the other independent influence factor for the effectiveness of colostomy on ulcerative CRP. ALB has commonly been used as a nutritional assessment indicator. It was reported that ALB was a simple biomarker of wound healing in patients suffering from ulcers [36–38]. As consistent with previous researches, our study also indicated that a higher level of ALB was facilitated to ulcer healing. In addition, if levels of Hb and RBC were reduced, decreased oxygen carrying capacity would occur, leading to tissue stress tolerance decrease and healing of pressure ulcer delay [39, 40]. In accordance with previous studies, significant differences were also found between the effective and ineffective groups in Hb and RBC levels. Additionally, it was reported that argon plasma coagulation (APC) procedure and almagate compound enema with NSAIDs were validated modality in the management of haemorrhagic radiation proctitis but may lead to chronic rectal ulcers [41, 42]. In this study, no significant difference was observed between the two groups in the application of APC and the almagate compound enema after colostomy for the included patients. Therefore, the baseline characteristics of the included patients in both groups are almost comparable.

## Conclusion

In conclusion, the overall effective rate of colostomy on ulcerative CRP was 49.2%, with a highest effective rate of 88.2% reached within 12 to 24 months after colostomy. 31.1% patients with ulcers were cured and 19.7% restored intestinal continuity, among which including 3.3% ever with rectovaginal fistula. The study demonstrated that colostomy was an effective and safe method for treating ulcerative CRP, and a potential strategy for preventing and treating fistula. Moreover, the duration of stoma and ALB level post-colostomy were independently associated with the effectiveness of colostomy on the rectal ulcer of CRP patients. However, our research was limited by the retrospective design and small sample size. A prospective study with greater sample size will be conducted to confirm our findings and further investigate the choice of surgery opportunity of colostomy for ulcerative CRP patients.

## Data Availability

All datasets used and/or analyzed during the current study are available from the corresponding author on reasonable request.

## Abbreviations

RE: Radiation enteritis
CRP: Chronic radiation proctitis
VRS: Vienna Rectoscopy Score
BMI: Body mass index
APC: Argon plasma coagulation
Hb: Hemoglobin
RBC: Red blood cells
BPC: Platelet count
ALB: Albumin
WBC: White blood cells
